# Study Conditions and University Students’ Mental Health during the Pandemic: Results of the COVID-19 German Student Well-Being Study (C19 GSWS)

**DOI:** 10.3390/ijerph20075286

**Published:** 2023-03-28

**Authors:** Eileen Heumann, Jannis Trümmler, Christiane Stock, Stefanie M. Helmer, Heide Busse, Sarah Negash, Claudia R. Pischke

**Affiliations:** 1 Institute of Health and Nursing Science, Charité—Universitätsmedizin Berlin, Corporate Member of Freie Universität Berlin and Humboldt-Universität zu Berlin, Augustenburger Platz 1, 13353 Berlin, Germany; 2 Institute of Medical Sociology, Centre for Health and Society, Medical Faculty, Heinrich-Heine-University Duesseldorf, 40225 Duesseldorf, Germany; 3 Unit for Health Promotion Research, University of Southern Denmark, Degnevej 14, 6705 Esbjerg, Denmark; 4 Institute for Public Health and Nursing Research, University of Bremen, 28359 Bremen, Germany; 5 Department Prevention and Evaluation, Leibniz Institute for Prevention Research and Epidemiology—BIPS, 28359 Bremen, Germany; 6 Institute for Medical Epidemiology, Biometrics and Informatics, Interdisciplinary Center for Health Sciences, Medical School of the Martin-Luther University Halle-Wittenberg, 06112 Halle (Saale), Germany

**Keywords:** university students, depressive symptoms, anxiety, mental health, study conditions, COVID-19 pandemic, counselling services

## Abstract

University students are generally vulnerable to mental health problems. This was exacerbated during the COVID-19 pandemic, when students experienced decisive changes and restrictions in their academic lives. Our study aimed at (a) analysing associations between study conditions and symptoms of depression and anxiety and (b) determining the extent of use and motivation to use student counselling services. The C19 GSWS is a cross-sectional study conducted at five universities in Germany (*N* = 7203). Descriptive analyses and linear regression models were performed to estimate the associations between study conditions and mental health outcomes. A total of 42.4% of the students felt down, depressed, or hopeless on several days over the past 14 days. Between a third and 44.1% of the students felt burdened by their study conditions. Worse perceived study conditions were associated with higher levels of depressive symptoms and anxiety. Only 7.1% indicated that they had utilised student counselling services, and female gender, enrolment in a bachelor’s programme, and having more than 1 reason for utilisation were factors associated with use. The results of our research underline the need for universities to review their study conditions and to provide targeted intervention strategies and counselling services to promote students’ mental well-being.

## 1. Introduction

University students are a population that is vulnerable to mental health problems (e.g., anxiety and depressive symptoms) [1], as they face developmental demands and formative life changes during the transition from late adolescence to emerging adulthood [2]. These challenges can cause uncertainty and may be perceived as a burden, which can impact on students’ mental health condition [3]. Stressors associated with studying, such as financial difficulties, academic performance, pressure to succeed, and post-graduation plans, make this population particularly vulnerable to depressive symptoms and anxiety [4]. Compared to individuals of the same age who are not studying at higher education institutions, first year university students are less likely to develop mental disorders, such as depression [5], or experience suicidal thoughts [6], but still experience high prevalence rates of affective disorders [5]. Furthermore, advanced students seem to be more affected than freshmen [4].

In addition to these challenges, the COVID-19 pandemic, with its ongoing restrictions in the public and academic realm, represents another multidimensional factor that further threatens students’ mental health [7]. Previous qualitative research conducted in Germany indicates that the change in teaching methods and learning behaviour due to the pandemic was considered as a serious burden [8]. Moreover, the reduced interaction with teachers and other students was perceived as a challenge by both students and teachers [9]. Further stressful experiences include the increased demands regarding self-learning skills, the subjectively perceived increased time spent studying due to the switch to online teaching and the fear of not being able to successfully complete the studies [10,11]. Furthermore, many university students experienced a worsened financial situation during the COVID-pandemic compared to the time before the COVID-19 pandemic, amplifying stress levels [12].

These COVID-19-related stress factors are found to be associated with the occurrence of mental health problems, resulting in higher prevalence rates for depressive symptoms and anxiety compared to pre-pandemic times [13]. Considering the results of a longitudinal study from a German university, the degree of self-reported loneliness was associated with pandemic-related factors, indicating that pandemic-related stress might have also contributed indirectly to mental health problems, such as depressive symptoms and anxiety [14]. It might have also exacerbated pre-existing psychological symptoms [15]. Hence, the lack of social interaction played an important role regarding the occurrence of depressive symptoms among university students during the pandemic [16].

Mental health problems in university students are associated with poor academic achievements [17] and an increased risk for university dropout [18]. As an important protective factor for mental health and life satisfaction [16,19], as well as academic performance [20], efforts to increase social support for students are required. Such support can also be offered by universities through adapted forms of counselling during the pandemic [11]. University students previously reported that using counselling services helped them maintain their academic performance and stay enrolled in their respective programs [21,22].

In this regard, our study follows up on the call by previous research suggesting a continuation of the examination of students’ perceptions of study conditions, mental health, and the use of university counselling services [11]. Our study contributes to this important public health task of monitoring students’ mental health with research on university students’ perceptions of their study conditions and their associations with mental health outcomes. Hence, our study aims (a) to identify study conditions that may be associated with depressive symptoms and anxiety within the study population and (b) to analyse the utilisation of student counselling services at universities and to explore the reasons for use and the predominant user groups.

## 2. Methods

### 2.1. Survey and Recruitment

The data of this study were assessed in the COVID-19 German Student Well-being Study (C19 GSWS). The C19 GSWS was based on and followed the cross-sectional COVID-19 International Student Well-being Study (C19 ISWS), which was conducted internationally at the beginning of the COVID-19 pandemic [23]. More information about the C19 GSWS study design is provided elsewhere [24].

In the C19 GSWS study, the questionnaire of the C19 ISWS study was used and adapted to the German university context. Moreover, further questions, e.g., regarding the vaccination status against COVID-19, were added. The final questionnaire contained 58 questions with different question types (45% single or multiple choice questions, 38% questions based on Likert scales, 10% questions with ranking, and 7% questions using an open format). The web-based survey was conducted from 27 October to 14 November 2021 via Limesurvey at five universities in Germany. The participating universities included the Heinrich-Heine-University Duesseldorf, the Charité—Universitätsmedizin Berlin, the Martin-Luther-University Halle-Wittenberg, the University of Bremen, and the University of Siegen. These universities had already participated in the preceding C19 ISWS and were, therefore, chosen for this second survey on students’ health and well-being at a later stage of the pandemic in Germany.

The participants were invited via email and e-learning platforms or via social media, such as Instagram. Before starting the survey, all students gave their informed consent to participate. The ethics committees of the five universities granted ethical approval for the study (University of Bremen 2021-28-EIL, University Halle-Wittenberg 2020-066, Heinrich-Heine-University 2020-958_1).

### 2.2. Study Sample and Context of the C19 GSWS

For data cleaning purposes, students who indicated that they were studying at a university other than the five universities mentioned above were excluded. Furthermore, all variables that went into statistical analyses were checked, and improbable values were removed, if necessary. Participants who filled out less than the first five pages of the online questionnaire or stated at the end of the survey that they had not conscientiously completed the questionnaire, but just wanted to have a look at the survey and completed the questionnaire at random, were excluded from the analysis. Data cleaning was done by two authors (EH and SMH). Subsequently, the data of 7203 students were included in the analysis. In total, 30.1% of the participants were enrolled at the Martin-Luther-University Halle-Wittenberg, 25.3% at the University of Bremen, 21.7% at the University of Siegen, 15.7% at the Charité—Universitätsmedizin Berlin, and 7.2% at the Heinrich-Heine-University Duesseldorf.

The survey was conducted during a time of an increasing COVID-19 incidence rate in Germany. From 27 October to 15 November 2021, the 7-day-incidence (new cases/100,000 citizens) of the total population in Germany rose by approximately 258% [25]. During the time of the survey, most universities conducted at least partial face-to-face teaching [25].

### 2.3. Measures

#### 2.3.1. Depressive Symptoms

We used the Centre for Epidemiological Studies Depression Scale (CES-D 8) to assess the frequency and severity of depressive symptoms [26]. The scale consists of eight items to assess how often during the last week (1) they felt depressed, (2) everything was an effort, (3) they slept restlessly, (4) could not get going, (5) felt lonely, (6) felt sad, (7) enjoyed life, and (8) felt happy. Students were asked to respond on a four-point Likert scale ranging from (0) ‘none or almost none of the time’, (1) ‘some of the time’, (2) ‘most of the time’ to (3) ‘all or almost all of the time’. We then calculated a continuous score, with a higher score indicating higher levels of depressive symptoms (score ranging from 0 to 24). Since there is no established and validated cut-off value for the CES-D 8 for university students, we decided to include this variable as a continuous score.

In addition to the CES-D 8 scale, we used the Patient Health Questionnaire (PHQ-2), a short version of the PHQ-9, for our analysis [27]. The PHQ-2 consists of the two first items of the PHQ-9 [27]. The stem question of the two items of the PHQ-2 is: ‘Over the last two weeks, how often have you been bothered by the following problems?’. The first item of the PHQ-2 is: ‘Feeling down, depressed or hopeless’ and the second is ‘little interest or pleasure in things’ including the following response options: (0) ‘not at all’, (1) ‘several days’, (2) ‘more than half the days’, and (3) ‘nearly every day’. We generated a score that summarized the two items, and the score ranged from 0 to 6, with a higher score indicating higher subjective depressive symptoms [27]. For descriptive analysis, we dichotomised the PHQ-2 score with a cut-off point of 3 (no depressive symptoms/depressive symptoms), according to Kroenke et al. [28].

The CES-D 8, as well as the PHQ-2, are standardized and validated survey instruments to assess depressive symptoms that are widely used in social sciences research [26,27,28,29]. They were used in our survey because they warrant comparability of our data with data gathered in other studies. Further, they were used in the preceding C19 ISWS study.

#### 2.3.2. Anxiety Symptoms

The General Anxiety Disorder-2 scale (GAD-2) is a valid and reliable scale to assess generalised anxiety symptoms [30]. It is widely used in social sciences research. This tool was used to ensure the comparability of our data with data gathered in other studies. Moreover, it was used in the preceding C19 ISWS study.

Respondents were asked: ‘Over the last two weeks, how often have you been bothered by the following problems?’ with respect to the items ‘feeling nervous, anxious, or on edge’ and ‘not being able to stop or control worrying’ and the same response options as for the PHQ-2 [30]. The score of the GAD-2 scale was computed in the same way as the PHQ-2 and also ranges from 0 to 6. A higher GAD-2 score indicates more anxiety symptoms. For descriptive analysis, we dichotomised the GAD-2 score with a cut-off point of 3 (no anxiety/anxiety) [30,31].

#### 2.3.3. Perceived Study Conditions

To assess the study conditions, we used the following items: (1) ‘My university/college workload has significantly increased since the COVID-19 outbreak’, (2) ‘I know less about what is expected of me in the different course modules/units since the COVID-19 outbreak’, (3) ‘I am concerned that I will not be able to successfully complete the academic year due to the COVID-19 outbreak’, (4) ‘The university/college provides poorer quality of education during the COVID-19 outbreak as before’, (5) ‘The change in teaching methods resulting from the COVID-19 outbreak has caused me significant stress’, and (6) ‘I feel I can talk to a member of the university/college staff (e.g., professor, student counsellor) about my concerns due to the COVID-19 outbreak’. Responses ranged from 1 ‘Strongly agree’ to 5 ‘strongly not agree’ on a five-point Likert scale. Two further variables were not considered because these two variables did not query the perceived study condition. For the analyses, we recoded the items 1 to 5 such that a higher Likert score indicates a higher level of academic stress due to the perceived study conditions. The perceived study conditions were summed up to an overall score. Afterwards, the mean was calculated, ranging from 1 to 5, with a higher score indicating higher levels of academic stress due to the perceived study conditions.

#### 2.3.4. Utilisation of Study Counselling

Students were asked the following questions: ‘Since the COVID-19 outbreak, did you seek contact with student counselling services or social services at your university/college?’, with the response options ‘yes’ or ‘no’. They were also asked for what reason they contacted counselling services, with the response options ‘discuss worries about studies’, ‘discuss financial worries or difficulties’ or ‘discuss psychosocial problems’, ‘discuss other worries, please specify’, or ‘prefer not to say’. For data analysis, if more than one answer was given, the answers were collapsed into the category ‘more than one answer’.

### 2.4. Data Analysis and Covariates

We performed a descriptive analysis (absolute, %) to summarise the sample in terms of sociodemographic data and further relevant information, such as relationship status or living situation. Further, descriptive analyses (absolute, %) of the items of the CES-D 8, PHQ-2, and GAD-2 and study conditions were performed. Moreover, we analysed the distribution between the utilisation of student counselling (yes or no) and other factors, such as sociodemographic characteristics and depressive (PHQ-2) and anxiety symptoms (GAD-2). We assume that there is a relationship between worse perceived study conditions and depressive symptoms, as well as anxiety. We, therefore, conducted three linear regression models to determine the associations between study conditions and depressive symptoms, as well as anxiety. The models included (1) the CES-D 8 scale, (2) the PHQ-2, and (3) the GAD-2 as dependent variables and the sum score of perceived study conditions as the independent variable.

The following covariates were also included in all statistical models: age (continuous), gender (female, male, diverse), relationship status (single, in a relationship, it is complicated), availability of a person to discuss intimate matters with (yes, no), study programme (bachelor, master, state examination, PhD), residency status in Germany (temporary, permanent), and living situation (shared household, alone). The formulas of all three regression models can be found in the Supplementary Materials S1. Before computing the three regression models, we checked the validity of method assumptions (linearity, no multicollinearity, homoscedasticity, normally distributed residuals) (Supplementary Materials S2).

Our methodological approach was based on a previous study by Matos Fialho et al. [11] to ensure external validation. This study used the 2020 C19 ISWS survey data to assess the association between the perceived study conditions and depressive symptoms (CES-D 8) with linear regression and descriptive statistics. We also considered some of the covariates without variable transformation in our models, as did Matos Fialho et al. [11].

## 3. Results

### 3.1. Sample Characteristics

The characteristics of the study sample are shown in Table 1. After data cleaning, a total of 7203 participants were included in the final data analysis. The average age of university students was 24.1 years (SD 5.0). Overall, 67.0% of the participants were female, and 46.1% were enrolled in a bachelor’s programme. Further details of the study sample are described in Table 1.

### 3.2. Depressive Symptoms and Anxiety

In Table 2, the percentages of students with depressive symptoms assessed with the CES-D 8 are shown. The mean of the CES-D 8 scale was 9.4 (SD 4.9). In Table 3, the percentages of students with depressive symptoms measured with the PHQ-2 are displayed. According to the PHQ-2, the mean score is 2.0 points (SD 1.6). When using the cut-off, 28.6% of the students were categorized as having depressive symptoms.

The results of the GAD-2 are shown in Table 4. The mean score of the GAD-2 was 2.0 (SD 1.7), and 31.2% of the students indicated anxiety symptoms.

### 3.3. Description of Study Conditions

In Table 5, the description of the characteristics of the study conditions are displayed. Approximately 2 in 5 students (39.7%) agreed with the statement that the workload had significantly increased since the COVID-19 outbreak. Almost half of the students (44.1%) agreed that they knew less of what was expected in the study modules. More than a quarter (28.6%) of the students were concerned that they would not successfully complete the academic year. More than half (55.1%) were not concerned about not being able to successfully complete the academic year. About 38% stated that their universities provided a poorer quality of education since the pandemic. Almost two-fifths of the students (38.8%) reported that the changes of teaching methods due to COVID-19 caused significant stress. Around two-thirds (65.9%) of the students stated that the universities did not sufficiently inform them regarding implemented changes due to COVID-19. About half of the students (52.8%) were satisfied with the way of implementing protective measures by the universities. About 38% stated they could not talk to a member of the university about their concerns. The mean of the study condition scale was 3.1 (SD 0.8).

### 3.4. Utilisation of Student Counselling

In Table 1, the utilisation of student counselling is displayed. Of all participants, 7.1% had contact with student counselling during the pandemic. Overall, 111 (24.6%) and 101 (22.4%) students used counselling services to discuss their worries or psychosocial problems, respectively. A total of 35.7% (161 participants) had more than 1 reason for contacting student counselling, and 5.5% (25 participants) wanted to discuss financial worries or difficulties.

Table 6 shows the utilisation of student counselling by further factors. Almost all students (90.0%) who utilised student counselling indicated that they have someone to discuss intimate matters with. Students who utilised student counselling were on average older compared to students who did not utilise student counselling (25.1 years vs. 24.0 years). The proportion of female students who utilised student counselling was higher (72.2%) compared with male and diverse students. Most students who utilised student counselling were enrolled in a bachelor programme (51.7%). Almost half (49.2%) of the students who utilised student counselling were in a relationship.

The analysis of the PHQ-2 revealed that almost half of the students (44.7%) who utilised student counselling also reported depressive symptoms. The GAD-2 revealed that 45.1% of the students who utilised student counselling services had anxiety symptoms.

### 3.5. Associations between Study Conditions, as Well as Further Determinants and Depressive Symptoms

Regarding the adjusted R^2^, the CES-D 8 model had a value of 0.2, and the PHQ-2 model had a value of 0.1. The ANOVA indicated for both models a significant result (*p* < 0.001) and an F-value of 109.1 for the CESD-8 model and 74.3 for the PHQ-2 model. According to the residual statistics, the Kolmogorov–Smirnov test of normality of the standardized residuals revealed a significant result (*p* < 0.001) for the CES-D 8 and the PHQ-2 model. Regarding the Durbin–Watson test for autocorrelation of the residuals, the test revealed a value of 2.0 for the CES-D 8, as well as the PHQ-2 model, and therefore, there is no autocorrelation. Regarding the Cronbach’s Alpha, we obtained a value of 0.86 for the CES-D 8 and 0.79 for the PHQ-2 in our sample.

The linear regression to analyse the association between the depressive symptoms and the study conditions showed that students perceiving worse study conditions had higher CES-D 8 and PHQ-2 scores (Table 7). Students who utilised student counselling showed higher levels of depressive symptoms (CES-D 8 and PHQ-2), compared to students who did not. Those students who indicated having no one with whom to discuss intimate matters with showed higher levels of depressive symptoms (CES-D 8 and PHQ-2), compared to those who had someone to discuss intimate matters with. Being male was associated with a lower CES-D 8, as well as PHQ-2, score, compared to female students. Moreover, students enrolled in master and state examination programmes were less likely to report depressive symptoms (CES-D 8 and PHQ-2), compared to students in a bachelor programme.

### 3.6. Associations between Study Conditions, as Well as Further Determinants and Anxiety Symptoms

Regarding the adjusted R^2^, the GAD-2 model had a value of 0.1. The ANOVA indicated a significant result (*p* < 0.001) and an F-value of 68.9. According to the residual statistics for the GAD-2 model, the Kolmogorov–Smirnov test of normality of the standardized residuals revealed a significant result (*p* < 0.001). Regarding the Durbin–Watson test for autocorrelation of the residuals, the test revealed a value of 2.0 for this model and, therefore, no autocorrelation. The Cronbach’s Alpha for the GAD-2 score was 0.78.

As shown in Table 8, the linear regression model revealed that worse perceived study conditions were associated with higher GAD-2 anxiety scores. University students who utilised student counselling services showed higher levels of anxiety symptoms, compared to those who did not. Students who reported not having anyone to discuss intimate matters with showed higher perceived anxiety symptoms than students who reported having someone to discuss intimate matters with. Male students had a lower GAD-2 score, compared to female students. On the other side, diverse students had a higher GAD-2 score, compared to female students. Moreover, students enrolled in master and state examination programmes were less likely to report anxiety symptoms, compared to students in a bachelor programme.

## 4. Discussion

This study focused on university students’ perceptions of study conditions and their associations with mental health outcomes. Moreover, the study aimed at assessing the utilisation of student counselling services. Between a third and slightly less than half of the students perceived a burden because of changed study conditions during the COVID-19 pandemic. In addition, our results suggest that higher academic stress and dissatisfaction were associated with poorer mental health outcomes. Only a few university students indicated that they had utilised counselling services, most of whom were female.

The participants in our sample perceived their study conditions overall as better compared with the previous study by Matos Fialho et al. [11] using the same questionnaire and study design. The present study is based upon the C19 ISWS survey conducted in 2020, but involves presumably not the same university students. However, four out of five universities participated in the first study, as well, which makes both samples quite comparable. This comparison can be made because of the very similar study characteristics (e.g., gender distribution or proportions in degree programmes) of the C19 ISWS and the C19 GSWS. An improvement in student ratings regards their study conditions during the pandemic is also supported by further evidence from earlier phases of the pandemic [32]. This could be explained by the fact that the university students had already gained experience with pandemic-related restrictions, which could have led to the development of coping strategies and behaviours [33,34]. Further, research suggests that university students handled the changed learning situation pragmatically, and some appreciated the increased amount of personal responsibility and independent working, while others reported difficulties concentrating and maintaining their motivation [35]. In this case, support from the university provided by lecturers and the administration can play a mediating role in the relationship between the perceived pressure to succeed at studying and mental well-being, as indicated in another study [36].

Regarding their mental health, students reported a higher level of depressive symptoms compared with our previous data collection in the first phase of the pandemic in 2020 [11]. Five of eight items on the CES-D 8 scale were rated worse. In comparison, a slightly higher percentage of university students stated that everything they did was an effort (29.7% vs. 35.0%; +5.3%) and that their sleep was restless (28.6% vs. 31.3%; +2.7%) in this study as compared to the previous one. These findings suggest that university students are affected long-term by similar or even higher levels of depressive symptoms, given that the samples stem from the same universities and are, therefore, to a certain extent comparable. A general deterioration in mental health and an increase in anxiety and depressive symptoms among university students during the pandemic was also found in other studies in Germany and internationally [14,37,38,39].

On the other hand, slightly more participants reported that they enjoyed life (42.0% vs. 46.4%; +4.4%) and felt happy compared to the previous study (50.6% vs. 52%; +1.4%) [11]. The higher level of happiness and life satisfaction and concurrent depressive symptomatology is difficult to interpret, but could be explained by the decreasing number of COVID-19 restrictions and their impact on everyday life in an overall and persistently stressful pandemic situation at the time of the data collection.

Our main findings regarding the associations between perceived worse study conditions and poorer mental health outcomes are in line with Matos Fialho et al. [11]. In addition, Plakhotnik et al. [35] found that worries about successful completion were associated with poor student well-being, indicating that concerns for future career opportunities affect student well-being over time. Qualitative research showed that study worries can lead to anxiety and doubts about completing a programme, to changes in the subject, or to dropping out [40]. In fact, research suggests that mental health problems during the pandemic led to higher drop-out rates than prior to the pandemic [41].

Social support seems to be a protective factor for depressive symptoms and anxiety in university students [42]. Our findings underline previous results, as our linear regression models indicated that university students who could not discuss intimate matters with someone else displayed more mental health symptoms. Social support during the pandemic could have also been offered by university counselling services. Yet, despite the increase in depressive symptoms and anxiety, the rate of help-seeking by contacting student counselling in our sample was still quite low (7.1%). A Portuguese study on help-seeking behaviours among university students with mental health problems during the pandemic also reported a significant increase in clinical symptomatology, while help-seeking behaviours did not change accordingly [43]. Previous evidence suggests that many university students do not seek help due to barriers, such as stigma and embarrassment [44,45]. However, student counselling services can be beneficial for university students’ academic success and partly for their mental health [21,22,46].

Nevertheless, the reasons for this contradiction should be further investigated, and the opportunity for low-threshold counselling in the university context should be expanded to meet the needs of university students. Therefore, it seems necessary to consider the burden of perceived study conditions when developing strategies to prevent and promote university students’ mental health.

### Strengths and Limitations

The multi-centre COVID-19 German Student Well-being Study provides evidence on associations between perceived study conditions and mental health among university students in Germany during the pandemic based on a large sample. Moreover, it contributes to the monitoring of usage patterns of university counselling services.

However, some limitations must be taken into account. Firstly, our analyses were based on a convenience sample, and more than a quarter of the participants were university students of health-related subjects or medicine. Thus, the results are not representative of the general German university student population. The sample was also gender imbalanced, with a larger proportion of female participants, and a selection bias cannot be ruled out.

Further, due to the cross-sectional design, it is not possible to draw conclusions about causality or changes in perception of study conditions, depressive symptomatology, anxiety, or student counselling utilisation over the duration of the pandemic or comparisons to pre-pandemic times. In addition, using self-assessed measures in the C19 GSWS survey may have resulted in response bias. To reduce this potential bias, our data were collected using a confidential online survey. Lastly, the negative phrasing of the study condition items may have induced a bias towards agreement with the problem description.

## 5. Conclusions

This study provided insights into perceived study conditions and associations with mental health outcomes among university students 20 months after the COVID-19 outbreak in Germany. It also provided information regarding the use and user groups of counselling services at universities during that time. Alongside other evidence, our study shows that university students are vulnerable to mental health problems. Study conditions are associated with mental health outcomes, such as anxiety and depressive symptoms. It is, therefore, necessary to examine the study conditions (e.g., considering the different degree programmes and study fields) more closely and to put a focus on the environmental level when designing health-promoting interventions in the university setting.

According to our results, the use of counselling services at universities is still low, despite the widespread pandemic- and health-related problems among university students. More in-depth qualitative research is needed to investigate university students’ counselling needs and the barriers for use. On the other hand, further research is required to assess university students’ competencies and resources, which can be strengthened with support measures. In conclusion, it is essential to consider both the environmental and individual level when designing future interventions aimed at preventing and promoting mental health in this population.

## Figures and Tables

**Table 1:** Participant characteristics (*n* = 7203).

Variables	*n* *	Mean (SD)
Age in years	7181	24.1 (SD 5.0)
	*n*	%
Gender		
Male	2199	30.6
Female	4824	67.0
Diverse	77	1.1
Degree programme		
Bachelor programme	3305	46.1
Master programme	1385	19.3
State examination (medicine, law)	2306	32.2
PhD	149	2.1
Relationship status		
In a relationship	3797	52.8
Single	2963	41.2
It is complicated	302	4.2
Residency status in Germany		
Permanent residency	6927	96.7
Temporary residency	238	3.3
Living situation		
Alone	1482	21.2
Shared living situation	5510	78.8
Utilisation of student counselling		
Yes	450	7.1
No	5911	92.9

SD: standard deviation; * differences in *n* are due to missing values.

**Table 2:** Depressive symptoms assessed with the CES-D 8 (*n* = 6848).

	None or Almost None of the Time		Some of the Time		Most of the Time		All or Almost All of the Time	
Variables	*n*	%	*n*	%	*n*	%	*n*	%
Felt depressed	1582	22.8	3542	51.1	1285	18.5	521	7.5
Everything was an effort	1499	21.6	3008	43.4	1715	24.7	710	10.2
Sleep was restless	1860	26.9	2898	41.9	1426	20.6	740	10.7
Happy	433	6.3	2891	41.8	2999	43.3	598	8.6
Felt lonely	2719	39.2	2785	40.2	976	14.1	448	6.5
Enjoyed life	732	10.6	2980	43.1	2563	37.0	647	9.3
Felt sad	1824	26.4	3694	53.4	1077	15.6	320	4.6
Could not get going	2197	31.8	2923	42.3	1259	18.2	531	7.7
	Mean	SD						
CES-D 8 score	9.4	4.9						

SD: standard deviation.

**Table 3:** Depressive symptoms assessed with the PHQ-2 (*n* = 6918).

	Not at All		Several Days		More Than Half the Days		Nearly Every Day	
Variables	N	%	N	%	N	%	N	%
Feeling down, depressed, or hopeless	2441	35.3	2933	42.4	1029	14.9	520	7.5
Little interest or pleasure in doing things	1716	24.8	3663	52.9	1074	15.5	470	6.8
	Mean	SD						
PHQ-2 score	2.0	1.6						

SD: standard deviation.

**Table 4:** Anxiety assessed with the GAD-2 (*n* = 6917).

	Not at All		Several Days		More Than Half the Days		Nearly Every Day	
Variables	*n*	%	*n*	%	*n*	%	*n*	%
Feeling nervous, anxious, or on edge	2013	29.1	3079	44.5	1175	17.0	655	9.1
Not being able to stop or control worrying	2868	41.4	2379	34.4	966	13.4	710	10.3
	Mean	SD						
GAD-2 score	2.0	1.7						

SD: standard deviation.

**Table 5:** Perceived study conditions among university students.

Variables	*n*	%
Workload has significantly increased since the COVID-19 outbreak		
Agree	2612	39.7
Neither agree nor disagree	2407	36.5
Disagree	1574	23.9
Know less what is expected of me in study modules		
Agree	2909	44.1
Neither agree nor disagree	1849	28.0
Disagree	1835	27.8
Concerned that I am not able to successfully complete academic year		
Agree	1897	28.6
Neither agree nor disagree	1080	16.3
Disagree	3657	55.1
The university provides poorer quality of education science the pandemic		
Agree	2469	37.5
Neither agree nor disagree	2108	32.1
Disagree	1995	30.4
Changes of teaching methods due to COVID-19 caused significant stress		
Agree	2557	38.8
Neither agree nor disagree	1570	23.8
Disagree	2462	37.4
I feel I can talk to a member of the university about my concerns		
Agree	1786	27.1
Neither agree nor disagree	2330	35.3
Disagree	2481	37.6
Study condition score (mean and SD)	3.1	SD 0.8

SD: standard deviation.

**Table 6:** Utilisation of student counselling by student characteristics.

	Utilisation of Student Counselling	
Variables	No (*n*, %)	Yes (*n*, %)
Person to discuss intimate matters with		
No	529 (9.6)	42 (10.0)
Yes	4985 (90.4)	378 (90.0)
Age in years (mean, SD)	24.0 (SD 4.8)	25.1 (SD 5.5)
Gender		
Male	1825 (30.9)	109 (24.2)
Female	3945 (66.8)	325 (72.2)
Diverse	65 (1.1)	6 (1.3)
Degree programme		
Bachelor programme	2696 (45.8)	231 (51.7)
Master programme	1132 (19.2)	93 (20.8)
State examination (medicine, law)	1924 (32.7)	116 (26.0)
PhD	119 (2.0)	5 (1.1)
Relationship status		
In a relationship	3123 (52.9)	221 (49.2)
Single	2448 (41.5)	185 (41.2)
It is complicated	230 (3.9)	33 (7.3)
Residency status in Germany		
Permanent residency	5701 (96.9)	428 (95.5)
Temporary residency	182 (3.1)	20 (4.5)
Living situation		
Alone	1214 (21.1)	105 (24.1)
Shared living situation	4544 (78.9)	331 (75.9)
PHQ-2		
No depressive Symptoms	4268 (72.4)	249 (55.3)
Depressive Symptoms	1630 (27.6)	201 (44.7)
GAD-2		
No anxiety symptoms	4126 (69.9)	247 (54.9)
Anxiety symptoms	1773 (30.1)	203 (45.1)

SD: standard deviation.

**Table 7:** Results of two linear regression models to determine the associations between depressive symptoms and study conditions, as well as further determinants.

	CES-D 8 (Dependent Variable)			PHQ-2 (Dependent Variable)		
Independent Variables	Reg. Coefficient	*t*-Value	95% CI	Reg. Coefficient	*t*-Value	95% CI
Problematic study conditions (metric)	1.9	24.0	1.7–2.1 ***	0.5	20.2	0.5–0.6 ***
Utilisation of student counselling						
No (ref.)	-		-	-	-	-
Yes	1.5	6.4	1.0–1.9 ***	0.4	5.7	0.3–0.6 ***
Anyone to discuss intimate matters with						
Yes (ref.)	-		-	-	-	-
No	3.7	17.8	3.2–4.0 ***	1.0	15.0	0.9–1.2 ***
Age	0.0 *	1.0	0.0 *–0.0 *	−0.01	−1.6	−0.0–0.0 *
Residency status in Germany						
Permanent residency (ref.)	-		-	-	-	-
Temporary residency	0.3	0.9	−0.4–1.0	0.2	1.5	−0.5–0.4
Living situation						
Living with others (ref.)	-	-	-	-	-	-
Alone	0.5	3.6	0.2–0.8 ***	0.05	1.1	−0.0 *–0.1
Gender						
Female (ref.)	-	-	-	-	-	-
Male	−1.0	−7.7	−1.2–(−0.7) ***	−0.2	−5.3	−0.3–(−0.1) ***
Diverse	1.0	1.8	−0.1–2.1	0.4	2.0	0.0 *–0.7 **
Relationship status						
In a relationship (ref.)	-	-	-	-	-	-
Single	0.4	3.3	0.2–0.7 ***	−0.0 *	−1.0	−0.1–0.0 *
It’s complicated	1.1	3.8	0.5–1.7 ***	0.2	2.0	0.0 *–0.4 **
Study programme						
Bachelor (ref.)	-	-	-	-	-	-
Master	−0.7	−4.1	−1.0–(−0.4) ***	−0.1	−2.6	−0.2–(−0.0 *) **
State examination	−1.4	−10.4	−1.6–(−1.1) ***	−0.4	−9.2	−0.5–(−0.3) ***
PhD	−0.8	−1.6	−1.6–0.1	−0.3	−2.1	−0.6–(−0.0 *)

* Due to rounding of results; CI: confidence interval; ** *p* < 0.05; *** *p* < 0.001.

**Table 8:** Results of the linear regression model to determine the associations between anxiety symptoms and study conditions, as well as further determinants.

	GAD-2 (Dependent Variable)		
Independent Variables	Reg. Coefficient	*t*-Value	95% CI
Problematic study condition (metric)	0.5	18.4	0.5–0.6 ***
Utilization of student counselling			
No (ref.)	-	-	-
Yes	0.6	7.1	0.4–0.7 ***
Anyone to discuss intimate matters			
Yes (ref.)	-	-	-
No	0.9	12.3	0.7–1.1 ***
Age	−0.0 *	−0.3	−0.0–0.0 *
Residency status in GER			
Permanent residency (ref.)	-	-	-
Temporary residency	0.2	1.4	−0.1–0.5
Living situation			
Living with others (ref.)	-	-	-
Alone	0.0 *	0.3	−0.1–0.1
Gender			
Female (ref.)	-	-	-
Male	−0.5	−11.0	−0.6–(−0.4) ***
Diverse	1.0	4.8	0.6–1.4 ***
Relationship status			
In a relationship (ref.)	-	-	-
single	−0.0 *	−0.3	−0.1–0.1
It’s complicated	0.2	1.5	−0.1–0.4
Study programme			
Bachelor (ref.)	-	-	-
Master	−0.2	−3.3	−0.3–(−0.1) **
State examination	−0.4	−7.1	−0.4–(−0.3) ***
PhD	−0.2	−1.3	−0.6–0.1

* Due to rounding of results; CI: confidence interval; ** *p* < 0.05; *** *p* < 0.001.

## Data Availability

The data set will be made available on Zenodo.

## Supplementary Materials

The following supporting information can be downloaded at: https://www.mdpi.com/article/10.3390/ijerph20075286/s1, S1: Formulas of the three regression models; S2: Validity of method assumptions check for the three regression models.
