# Correction: Heumann et al. Study Conditions and University Students’ Mental Health during the Pandemic: Results of the COVID-19 German Student Well-Being Study (C19 GSWS). *Int. J. Environ. Res. Public Health* 2023, *20*, 5286

**DOI:** 10.3390/ijerph21101280

**Published:** 2024-09-26

**Authors:** Eileen Heumann, Jannis Trümmler, Christiane Stock, Stefanie M. Helmer, Heide Busse, Sarah Negash, Claudia R. Pischke

**Affiliations:** 1 Institute of Health and Nursing Science, Charité—Universitätsmedizin Berlin, Corporate Member of Freie Universität Berlin and Humboldt-Universität zu Berlin, Augustenburger Platz 1, 13353 Berlin, Germany; 2 Institute of Medical Sociology, Centre for Health and Society, Medical Faculty, Heinrich-Heine-University Duesseldorf, 40225 Duesseldorf, Germany; 3 Unit for Health Promotion Research, University of Southern Denmark, Degnevej 14, 6705 Esbjerg, Denmark; 4 Institute for Public Health and Nursing Research, University of Bremen, 28359 Bremen, Germany; 5 Department Prevention and Evaluation, Leibniz Institute for Prevention Research and Epidemiology—BIPS, 28359 Bremen, Germany; 6 Institute for Medical Epidemiology, Biometrics and Informatics, Interdisciplinary Center for Health Sciences, Medical School of the Martin-Luther University Halle-Wittenberg, 06112 Halle (Saale), Germany

There was an error in the original publication [1]. After careful revision, we found an error in the methodological approach regarding the CES-D 8 score dichotomisation and its application in Table 6. We initially used the recommended cut-off of 16 for the CES-D 20, but our study used the CES-D 8. Applying this cut-off underestimated the prevalence of depressive symptoms as validation studies for the CES-D 8 suggest lower cut-offs of 8 or 9.

Given these discrepancies and the lack of a specific cut-off for university students, it is more appropriate to use the CES-D 8 on a continuous basis. Thus, we refrain from reporting the prevalence rate using this instrument. Consequently, the cross-table showing the binary variable for depressive symptoms and the utilisation of student counselling is incorrect.

A correction has been made to 2. Methods, 2.3. Measures, 2.3.1 Depressive Symptoms, first paragraph:

We used the Centre for Epidemiological Studies Depression Scale (CES-D 8) to assess the frequency and severity of depressive symptoms [26]. The scale consists of eight items to assess how often during the last week (1) they felt depressed, (2) everything was an effort, (3) they slept restlessly, (4) could not get going, (5) felt lonely, (6) felt sad, (7) enjoyed life, and (8) felt happy. Students were asked to respond on a four-point Likert scale ranging from (0) ‘none or almost none of the time’, (1) ‘some of the time’, (2) ‘most of the time’ to (3) ‘all or almost all of the time’. We then calculated a continuous score, with a higher score indicating higher levels of depressive symptoms (score ranging from 0 to 24). Since there is no established and validated cut-off value for the CES-D 8 for university students, we decided to include this variable as a continuous score.

A correction has been made to 2. Methods, 2.4. Data Analysis and Covariates, first paragraph:

We performed a descriptive analysis (absolute, %) to summarise the sample in terms of sociodemographic data and further relevant information, such as relationship status or living situation. Further, descriptive analyses (absolute, %) of the items of the CES-D 8, PHQ-2, and GAD-2 and study conditions were performed. Moreover, we analysed the distribution between the utilisation of student counselling (yes or no) and other factors, such as sociodemographic characteristics and depressive (PHQ-2) and anxiety symptoms (GAD-2). We assume that there is a relationship between worse perceived study conditions and depressive symptoms, as well as anxiety. We, therefore, conducted three linear regression models to determine the associations between study conditions and depressive symptoms, as well as anxiety. The models included (1) the CES-D 8 scale, (2) the PHQ-2, and (3) the GAD-2 as dependent variables and the sum score of perceived study conditions as the independent variable.

Another correction has been made to 3. Results, 3.2. Depressive Symptoms and Anxiety, first paragraph:

In Table 2, the percentages of students with depressive symptoms assessed with the CES-D 8 are shown. The mean of the CES-D 8 scale was 9.4 (SD 4.9). In Table 3, the percentages of students with depressive symptoms measured with the PHQ-2 are displayed. According to the PHQ-2, the mean score is 2.0 points (SD 1.6). When using the cut-off, 28.6% of the students were categorized as having depressive symptoms.

Another correction has been made to 3. Results, 3.4. Utilisation of Student Counselling, third paragraph, and Table 6:

The analysis of the PHQ-2 revealed that almost half of the students (44.7%) who utilised student counselling also reported depressive symptoms. The GAD-2 revealed that 45.1% of the students who utilised student counselling services had anxiety symptoms.

**Table 6:** Utilisation of student counselling by student characteristics.

	Utilisation of Student Counselling	
Variables	No (*n*, %)	Yes (*n*, %)
Person to discuss intimate matters with		
No	529 (9.6)	42 (10.0)
Yes	4985 (90.4)	378 (90.0)
Age in years (mean, SD)	24.0 (SD 4.8)	25.1 (SD 5.5)
Gender		
Male	1825 (30.9)	109 (24.2)
Female	3945 (66.8)	325 (72.2)
Diverse	65 (1.1)	6 (1.3)
Degree programme		
Bachelor programme	2696 (45.8)	231 (51.7)
Master programme	1132 (19.2)	93 (20.8)
State examination (medicine, law)	1924 (32.7)	116 (26.0)
PhD	119 (2.0)	5 (1.1)
Relationship status		
In a relationship	3123 (52.9)	221 (49.2)
Single	2448 (41.5)	185 (41.2)
It is complicated	230 (3.9)	33 (7.3)
Residency status in Germany		
Permanent residency	5701 (96.9)	428 (95.5)
Temporary residency	182 (3.1)	20 (4.5)
Living situation		
Alone	1214 (21.1)	105 (24.1)
Shared living situation	4544 (78.9)	331 (75.9)
PHQ-2		
No depressive Symptoms	4268 (72.4)	249 (55.3)
Depressive Symptoms	1630 (27.6)	201 (44.7)
GAD-2		
No anxiety symptoms	4126 (69.9)	247 (54.9)
Anxiety symptoms	1773 (30.1)	203 (45.1)

SD: standard deviation.

A slight clarification has been made to 1. Introduction:

Compared to individuals of the same age who are not studying at higher education institutions, first year university students are less likely to develop mental disorders, such as depression [5], or experience suicidal thoughts [6], but still experience high prevalence rates of affective disorders [5]. Furthermore, advanced students seem to be more affected than freshmen [4].

Furthermore, we wish to note that we refrain from citing Heumann et al. (2023), as they have decided to retract their article (former reference 5).

With this correction, the order of some references has been adjusted accordingly (e. g., former reference 37 is now reference 36, and The original reference 28 in the publication was moved two positions to the back). Furthermore, since we decided to delete parts of the previous methods section, the order of some references has changed. The new paragraphs are as follows: 

In addition to the CES-D 8 scale, we used the Patient Health Questionnaire (PHQ-2), a short version of the PHQ-9, for our analysis [27]. The PHQ-2 consists of the two first items of the PHQ-9 [27]. The stem question of the two items of the PHQ-2 is: ‘Over the last two weeks, how often have you been bothered by the following problems?’. The first item of the PHQ-2 is: ‘Feeling down, depressed or hopeless’ and the second is ‘little interest or pleasure in things’ including the following response options: (0) ‘not at all’, (1) ‘several days’, (2) ‘more than half the days’, and (3) ‘nearly every day’. We generated a score that summarized the two items, and the score ranged from 0 to 6, with a higher score indicating higher subjective depressive symptoms [27]. For descriptive analysis, we dichotomised the PHQ-2 score with a cut-off point of 3 (no depressive symptoms/depressive symptoms), according to Kroenke et al. [28].

The CES-D 8, as well as the PHQ-2, are standardized and validated survey instruments to assess depressive symptoms that are widely used in social sciences research [26,27,28,29]. They were used in our survey because they warrant comparability of our data with data gathered in other studies. Further, they were used in the preceding C19 ISWS study.

2.3.2. Anxiety Symptoms

The General Anxiety Disorder-2 scale (GAD-2) is a valid and reliable scale to assess generalised anxiety symptoms [30]. It is widely used in social sciences research. This tool was used to ensure the comparability of our data with data gathered in other studies. Moreover, it was used in the preceding C19 ISWS study.

Respondents were asked: ‘Over the last two weeks, how often have you been bothered by the following problems?’ with respect to the items ‘feeling nervous, anxious, or on edge’ and ‘not being able to stop or control worrying’ and the same response options as for the PHQ-2 [30]. The score of the GAD-2 scale was computed in the same way as the PHQ-2 and also ranges from 0 to 6. A higher GAD-2 score indicates more anxiety symptoms. For descriptive analysis, we dichotomised the GAD-2 score with a cut-off point of 3 (no anxiety/anxiety) [30,31]. 

A slight clarification has been made to 4. Discussion:

Previous evidence suggests that many university students do not seek help due to barriers, such as stigma and embarrassment [44,45].

The authors state that the scientific conclusions are unaffected. This correction was approved by the Academic Editor. The original publication has also been updated.

## References

[1] Heumann E., Trümmler J., Stock C., Helmer S.M., Busse H., Negash S., Pischke C.R. (2023). Study conditions and university students’ mental health during the pandemic: Results of the COVID-19 German student well-being study (C19 GSWS). Int. J. Environ. Res. Public Health.

