# Supplementary material for: Study Conditions and University Students’ Mental Health during the Pandemic: Results of the COVID-19 German Student Well-Being Study (C19 GSWS)
Source: Int J Environ Res Public Health. 2023 Mar 28;20(7):5286. doi: 10.3390/ijerph20075286 (PMC10094523; doi:10.3390/ijerph20075286)
Supplement: Supplementary file 1 [file ijerph-20-05286-s001.zip › ijerph-2141570-supplementary.pdf]

International Journal of
Environmental Research and Public Health

MDPI

Supplementary material

S1: Formulas of the three regression models

Formula of the CES-D 8 model

CES-D 8=$ß_1$ + study conditions X $ß_2$ + utilization of study conditions X $ß_3$ + anyone to discuss intimate matters with X $ß_4$ + age X $ß_5$ + residency status in Germany X $ß_6$ + living situation X $ß_7$ + gender X $ß_8$ + relationship status X $ß_9$ + study programme X $ß_{10}$ + ε

Formula of the PHQ-2 model

PHQ-2=$ß_1$ + study conditions X $ß_2$ + utilization of study conditions X $ß_3$ + anyone to discuss intimate matters with X $ß_4$ + age X $ß_5$ + residency status in Germany X $ß_6$ + living situation X $ß_7$ + gender X $ß_8$ + relationship status X $ß_9$ + study programme X $ß_{10}$ + ε

Formula of the GAD-2 model

GAD-2=$ß_1$ + study conditions X $ß_2$ + utilization of study conditions X $ß_3$ + anyone to discuss intimate matters with X $ß_4$ + age X $ß_5$ + residency status in Germany X $ß_6$ + living situation X $ß_7$ + gender X $ß_8$ + relationship status X $ß_9$ + study programme X $ß_{10}$ + ε

S2: Validity of method assumptions check for the three regression models

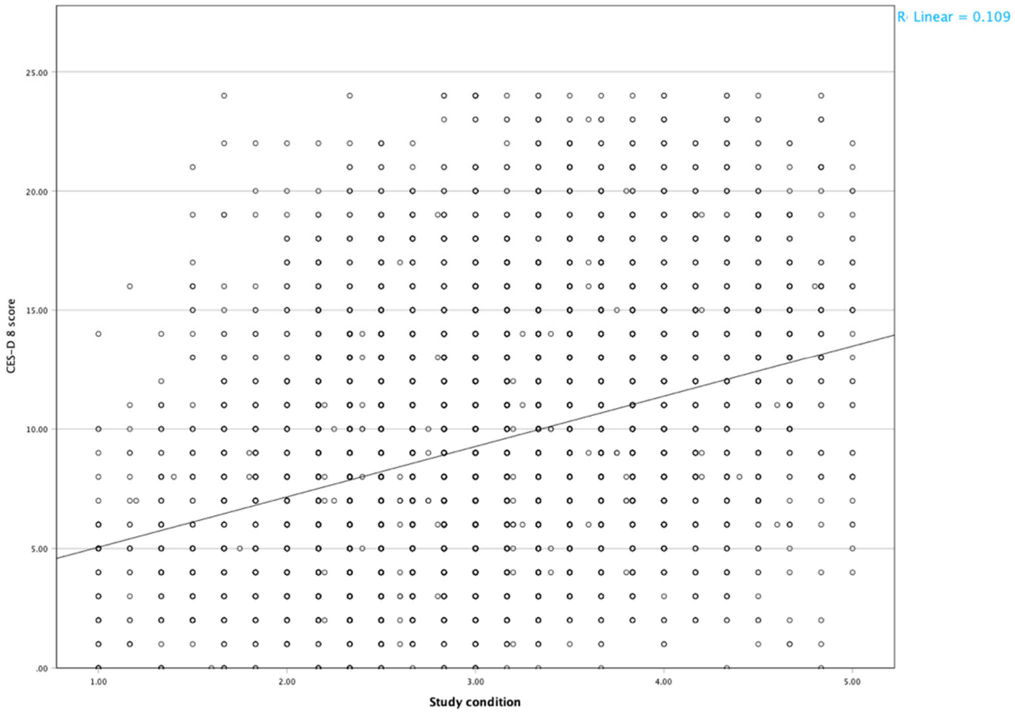

Figure S2.1: Linearity between the CES-D 8 score and study condition score

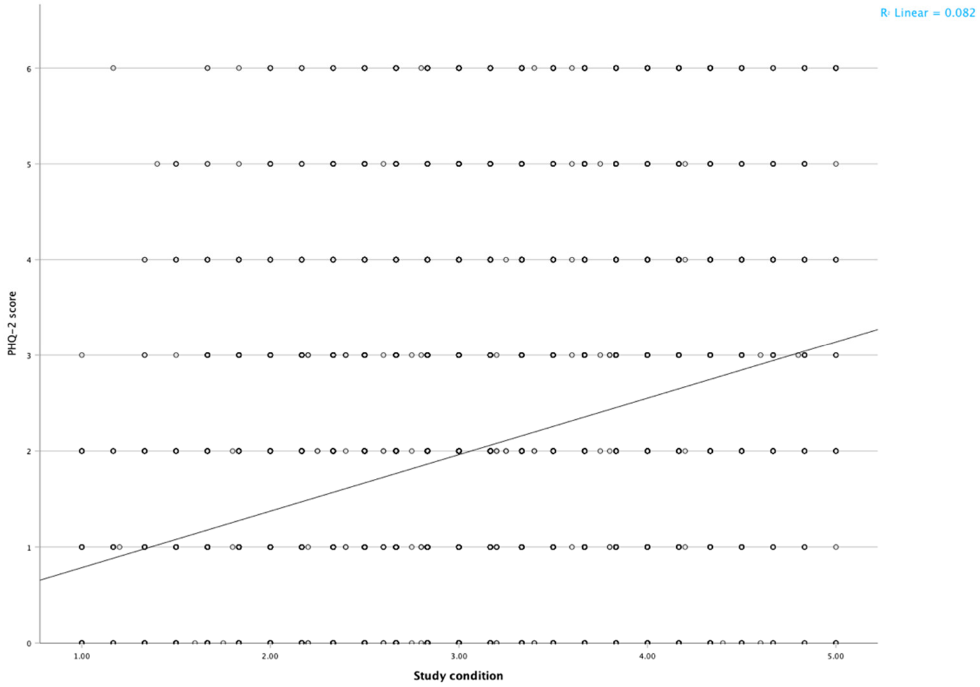

Figure S2.2: Linearity between the PHQ-2 score and study condition score

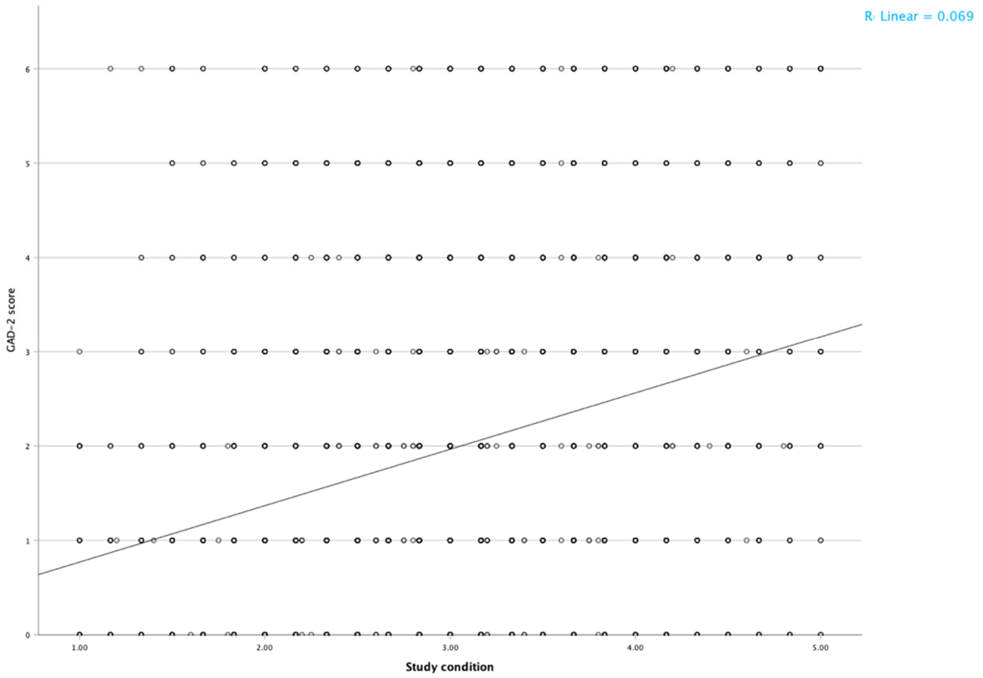

Figure S2.3: Linearity between the GAD-2 score and study condition score

Table S2.1: Test for multicollinearity between the dependent variables (CES-D 8, PHQ-2, GAD-2) and all independent variables

	CES-D 8		PHQ-2		GAD-2	
	**Tolerance**	**VIF**	**Tolerance**	**VIF**	**Tolerance**	**VIF**
**Problematic study conditions (metric)**	0.957	1.04	0.957	1.044	0.957	1.044
**Utilisation of student counselling**	0.980	1.02	0.980	1.020	0.980	1.020
**Anyone to discuss intimate matters with**	0.932	1.07	0.930	1.075	0.930	1.074
**Age**	0.856	1.168	0.856	1.167	0.856	1.167
**Residency status in Germany**	0.930	1.075	0.931	1.073	0.931	1.073
**Living situation**	0.949	1.053	0.951	1.051	0.951	1.051
**Male**	0.968	1.053	0.965	1.035	0.965	1.035
**Diverse**	0.992	1.008	0.991	1.008	0.991	1.008
**Single**	0.872	1.147	0.872	1.146	0.872	1.146
**It’s complicated**	0.951	1.050	0.951	1.050	0.951	1.050
**Master programme**	0.788	1.270	0.787	1.270	0.787	1.269
**Examination programme**	0.848	1.180	0.847	1.179	0.848	1.179
**PhD programme**	0.887	1.127	0.887	1.126	0.887	1.126

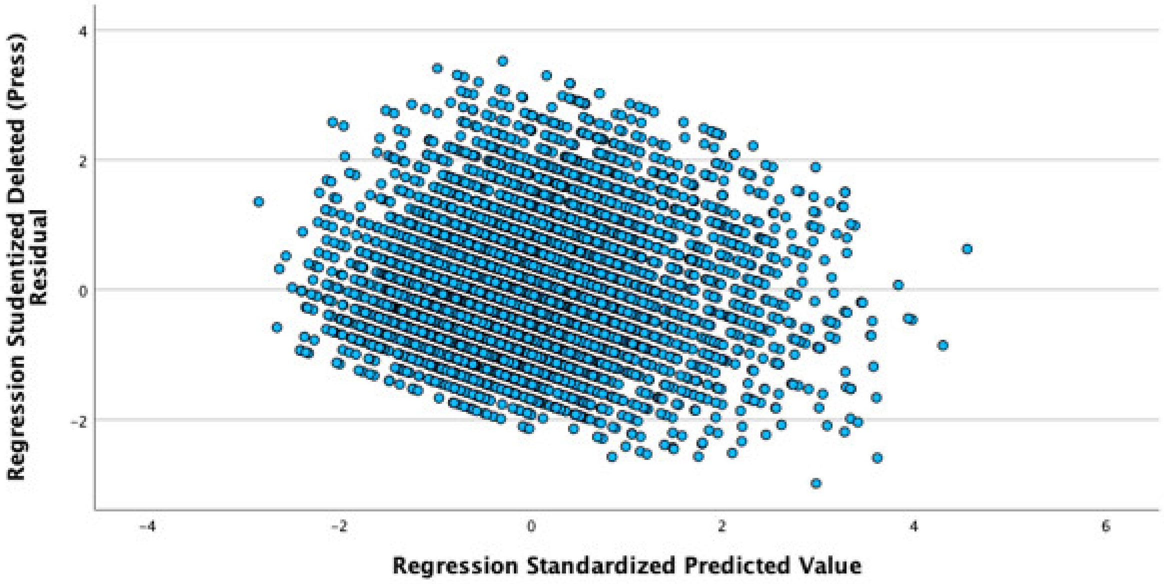

Figure S2.4: Homoscedasticity between CES-D 8 and all independent variables

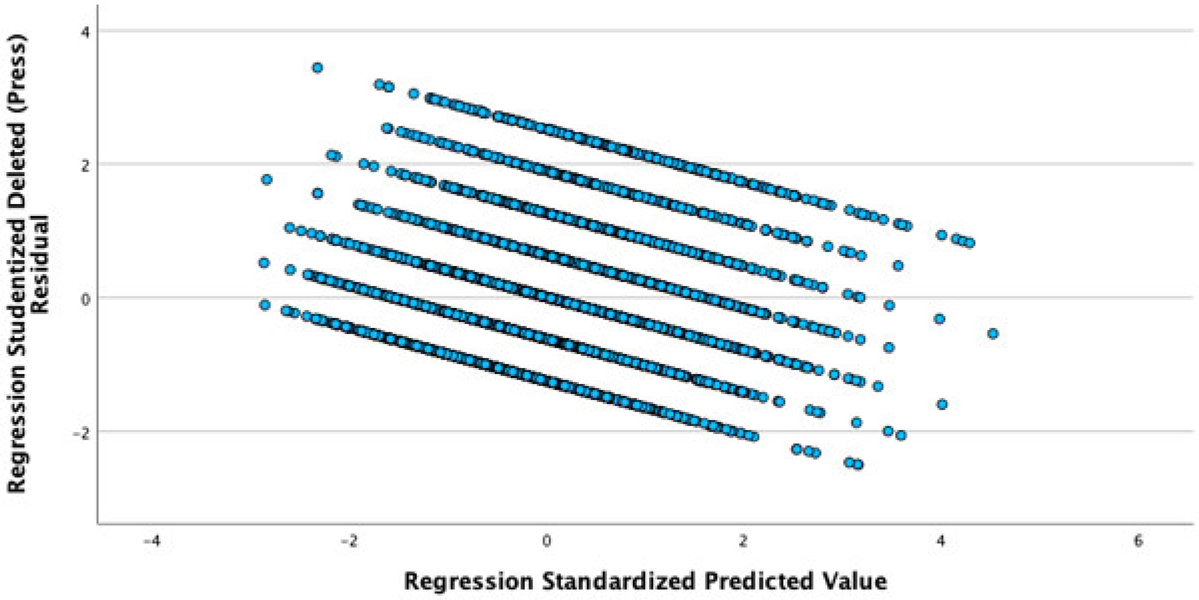

Figure S2.5: Homoscedasticity between PHQ-2 and all independent variables

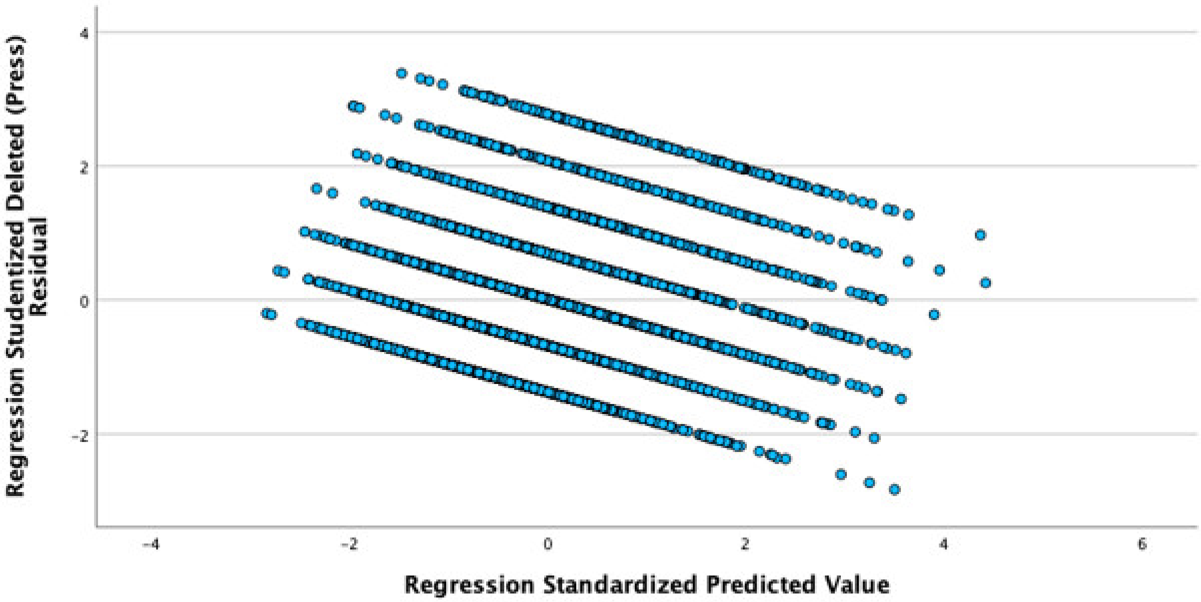

Figure S2.6: Homoscedasticity between the GAD-2 and all independent variables

Table S2.2: Kolmogorov-Smirnov test to check normal distributed residuals between the dependent variables (CES-D 8, PHQ-2, GAD-2) and all independent variables

	**Kolmogorov-Smirnov test**		
	**CES-D 8 (p-value)**	**PHQ-2 (p-value)**	**GAD-2 (p-value)**
**Unstandardized residuals**	<0.001	<0.001	<0.001
